# Modern Sociology

**Published:** 1896-12-12

**Authors:** 


### Modern Sociology.

### MISTAKEN IMPRESSIONS OF PRISON LIFE.—VI.

It seems almost like leading a forlorn hope in the battle for truth, to seek to combat the unfounded opinions which exist in this country on the subject of capital punishment, so deeply rooted are they in the minds of the public. The main points on which mistaken views are rife may be classified thus: First, that death on the gallows is the penalty which criminals dread the most; second, that it is a certain deterrent of crime; third, that it is the divinely-appointed punishment for murder; fourth, that persons desirous of seeing the death penalty abolished are actuated solely by a weak sentimentality and undue consideration for the criminal, instead of a just compassion for his victim, without having any substantial reasons for their ill-judged clemency. We are prepared to protest strongly against every one of these conclusions, and in the brief space at our disposal we may best do so by stating broad facts which tell most distinctly against them.

With regard to the first proposition, that death at the hands of the executioner is the punishment most dreaded by criminals, there seems to be already a somewhat sufficient proof that this cannot be the case, in the records of suicide, which of late has taken almost the form of an epidemic of self-destruction. It will be found that the great majority of persons who execute themselves do so by the same process as that employed legally, and hang themselves, only rather more clumsily than the manner in which the same proceeding is carried out by the judicial expert. But we have surer ground on which to base our absolute denial of the proposition, and that is by the testimony of men with whom we have conversed within a few days of their execution, and who have stated emphatically that they infinitely preferred a speedy death to a protracted term of penal servitude. Here are the very words uttered by one of these men in the open court where he was brought to trial for murder. "I wish to be hanged, sir—out of it—yes!" And it would be the statement of the great majority of criminals, because penal servitude and not death is the punishment they dread the most. We may add that we heard the same willing acceptance of the death penalty from the lips of a woman awaiting execution for murder. The second erroneous proposition, that capital punishment is a deterrent of crime, can be disproved by those who have had definite means of studying the subject in all its bearings. Certain statistics, with which we are acquainted, have shown distinctly that at the time when executions still took place in public, every one of these ghastly sights for many years back, had been witnessed by men and even by women, who afterwards underwent the same penalty for crimes subsequently committed; in more recent times; the significant hoisting of the black flag and the newspaper accounts of the murderer's doom have been eagerly scanned by those who were ultimately to share the same fate for the same crime—they had seen, and gloated over, all the gruesome details of the hangman's function, but it did not deter them for a moment from fatal acts of violence to which they were afterwards tempted. How should it, indeed! Murders are for the most part the result of some sudden fierce impulse of rage, roused into momentary frenzy by jealousy, or hatred, or revenge; and when the means of gratifying any of these master passions of the complex human nature are actually within their grasp, is it likely that the thought of a possible gallows would come into their minds at all, or that any consequence which might result from their indulgence in the overwhelming fury of the moment, would restrain them from it? In the case of more premediated murders, or those committed by burglars to conceal their identity, such persons are always strongly convinced that they will find it possible to escape detection, as, in fact, they very often succeed in doing. We cannot spare more space for proofs on this point, and therefore can only reiterate the assertion founded on the experience of many acute judges—that the death penalty is not a deterrent of crime.

The third erroneous opinion we have named, that it is the divinely-appointed punishment for murder, is founded on a single verse in the Book of Genesis, which, according to more careful translation, was in all probability not in any sense a command, but a prophecy. It was quite likely in these rude times that whoso shed man's blood would meet with the sure reprisals that by man his blood would be shed. In this view there is no trace of a Divine enactment which, indeed, could scarcely co-exist with the undoubted fact that under a Theocracy the first murderer, Cain, was sentenced, not to death, but to life, throughout the whole of which he would have to endure the natural consequences of his crime. There remaius our last indictment against public opinion in this connection, viz., that all who do not uphold capital punishment as a Divine and beneficent enactment are merely weakly benevolent persons, who are actuated by a foolish tenderness for the feelings of atrocious criminals. It may well be rather the sternest moralists desiring the most severe punishment possible for murderers, who would demur to their being only visited with the penalty which they dread much less than others that might be inflicted on them; but the objection to capital punishment by many wise thinkers, stands on far higher ground. Scientific progress in the present century has brought us a new revelation of the truth that life and death are alike unscrutable mysteries; of life we understand little enough, but of death we only know that it is impenetrable and irrevocable—in dealing it out to a fellow-mortal we are therefore inflicting a punishment of which we know neither the meaning, nor the issue nor the extent—is this justice? Within the limits of this tangible life his crime was committed, and within these limits only, can we know exactly what we are doing in the retribution with which we visit his sin on earth; a penalty to be endured by him in this life, can be weighed and measured in proportion to his crime on the most rigid lines of justice; but when we drive a man out of this world by the hangman's rope, we fling him out of our sight, out of our knowledge—we dismiss him from our responsibilities to a wholly unknown destiny. Have we any right also to inflict a punishment which is irrevocable, considering the fallibility of human judgment that has often under the *Lex taliones* executed those who were wholly innocent? These considerations might be greatly amplified, and under them it is a sense of justice and not a weak benevolence which actuates those who are intelligently opposed to capital punishment.

